# Optimal Imaging Strategies for Rectal Cancer Staging and Ongoing Management

**DOI:** 10.1007/s11864-016-0403-7

**Published:** 2016-06-02

**Authors:** Svetlana Balyasnikova, Gina Brown

**Affiliations:** Colorectal Imaging Group, The Royal Marsden Hospital, NHS Foundation Trust, Downs Road, Sutton, Surrey, SM2 5PT UK; Imperial College London, London, UK; The N.N. Blokhin Cancer Research Center, Kashirskoye Shosse 24, 15478 Moscow, Russia; The National Scientific Center of Coloproctology, ul. Saliama Adilia 2 123423 Moscow, Russia

**Keywords:** Rectal cancer staging, High-resolution MRI, Early and advanced rectal cancer, ERUS, EMVI, CRM, PET/CT, T stage, N stage, EMVI, Response assessment, Recurrent and mucinous rectal cancer

## Abstract

Imaging determines the optimal treatment for rectal cancer patients. High-resolution magnetic resonance imaging (MRI) overcomes many of the known limitations of previous methods. When performed in accordance with the recommended standards, MRI enables accurate staging of both early and advanced rectal cancer, accurate response assessment, the delineation of recurrent disease and planning surgical treatment in a safe and effective manner. Tumour-related high-risk features with known adverse outcomes can be preoperatively identified and treated with neoadjuvant chemoradiotherapy. Further, MRI post-treatment tumour response assessment using TRG grading system also predicts the likely survival outcomes and in the future will be used to modify treatment further by stratification into good and poor responders. There is a paucity of literature with validated outcome data concerning use of diffusion-weighted imaging and positron emission tomography (PET)/computed tomography (CT), and in the absence of any validated methods and outcome data, their use in the initial assessment and restaging after treatment is limited to research protocols. Combination MRI and CT is essential for distant spread assessment and recurrent disease, and currently PET-CT is sometimes used in the workup of patients with recurrent and metastatic disease.

## Introduction

Preoperative assessment of tumour spread has become essential for both early and locally advanced rectal cancer, response assessment to neoadjuvant chemoradiotherapy (CRT) and resectability of recurrent disease. A number of different imaging modalities have been used to assess locoregional and distant tumour spread, with magnetic resonance imaging (MRI) use predominating for rectal cancer staging in the last 10 years. Nowadays, for the majority of colorectal units, imaging assessment of the primary tumour helps to determine the surgical procedures and the necessity for preoperative treatment.

Implementation of bowel cancer screening programs has increased the rate of patient identified with early rectal cancers (ERC, defined as small early stage tumours, T1/T2 and polyps) [1] who could potentially benefit from adequately less aggressive surgical treatment; imaging is mandatory for extramural spread assessment and modern high-resolution methods can even be used to define the degree of preservation of submucosa and muscularis propria (provided that the imaging quality complies with published standards) [2]. Specific imaging features of tumour aggressiveness proven to predict outcomes such as magnetic resonance (MR)-circumferential resection margin (CRM) [3] and MR presence of extramural vascular invasion (EMVI) [4] should also be taken into consideration when treatment decisions are made in order to reduce risks of both local and distant failure in rectal cancer patients.

Imaging is becoming necessary for tumour response assessment when patients have completed neoadjuvant therapy. Although histopathology remains the so-called gold standard for evaluating tumour response within the final specimen, the opportunity to modify treatment before definitive surgery takes place is missed. Thus, preoperative identification of imaging complete responders (cr) will enable detection of patients who could potentially undergo organ-preserving treatment in the future and trials are underway to test the performance of MRI in the identification and surveillance of such patients. Conversely, preoperative detection of poor responders may lead to further consolidation therapy in order to reduce risks of local and distant failure. Thus, the post-treatment assessment of response using high-resolution MRI may in the future become a crucial step in the management of patients with advanced rectal cancer (TRIGGER: EudraCT Number 2015-003009-40).

Patients with locally recurrent rectal cancer form a challenging group for cure due to limited treatment option as CRT and surgery have usually been offered previously. The importance of accurate imaging has been recognised for these patients; a risk stratification staging system is based on number and location of pelvic compartments involved as identified on high-resolution MRI. This method of assessment has been shown to predict clinical and survival outcomes [5, 6•].

Imaging modalities involved in the local and distant staging of rectal cancer are the following: endorectal ultrasound (ERUS), MRI, computed tomography (CT) and positron emission tomography (PET)/CT (a summary of different methods performance is listed in a Table 1). Both ERUS and MRI are commonly recommended for ERC staging, and there is also strong agreement that MRI has become a required standard for evaluating locally advanced disease particularly those patients with potential CRM involvement. According to NCCN (North America) [7]/NICE (UK) [8] guidelines, contrast-enhanced CT of the chest, abdomen and pelvic should be offered to rectal cancer patients in order to estimate distant spread of the disease [8]. Excessive cost and time of a PET/CT scan limits its role to second choice modality when CT or MRI results are ambiguous in assessment of distant tumour spread. There are many discussions regarding the use of PET/CT for evaluating response to treatment; however, no consensus has been found in performing PET/CT in this context. Nevertheless, a combination of different modalities, including PET/CT, is almost always required in imaging of recurrent disease [9, 10].

**Table 1:** Advantages and disadvantages of different imaging methods.

Clinical indication	Imaging modality			
	ERUS	CT	MRI	PET/CT
Differentiation between benign polyps and invasive adenocarcinomas	+ Elastography appears promising	–	− No validated data	–
Early rectal cancer (T stage)	+ Only small T1sm1/2 tumours	–	+ Enables visualisation of submucosa and muscularis propria and substaging of T1 and identification of extramural disease	–
Advanced rectal cancer (T stage)	–	+	+ Allows T3 substaging	+ Only CT component
N stage	+/− Upper mesorectum and PSW compartments are not in the FOV	–	+	–
EMVI status	− No validated data	+	+ With greater sensitivity than histopathology, especially after chemoradiotherapy	+ Only CT component
CRM status	–	–	+ (Distance from tumour to the mesorectal fascia and intersphincteric plane ≤1 is considered as CRM involved)	–
Response assessment	− No validated data	–	+ (mr TRG predicts survival outcomes)	+/−
Recurrence	+/− Could be used in the limited number of cases (limitations: stenosing tumour, after APE or exenteration)	–	+ Enable defining extend of the disease within the pelvic compartments	+/−

+ accurate, +/− published data is controversial/application of the method is limited, – inaccurate/no validated data, *PSW* pelvic side wall

## ERUS

ERUS with the rigid probe has a number of imaging limitations including inability to stage high, bulky and structuring tumours [11]. Use of either a 7–10-MHz or flexible rigid ERUS ultrasound probe results in a small field of view (FOV) that prevents assessment of tumour relationship to the potential circumferential margin (CRM). Inability to distinguish peritumoral fibrosis from tumour spread limits use of ERUS in performing both primary and post-CRT assessment of rectal cancer [12]. The crucial limitation of ERUS assessment techniques is the limited views of the whole mesorectum and pelvis to exclude tumour deposits, discontinuous vascular invasion and mesorectal fascia involvement by tumour. Thus, disease beyond the immediate vicinity of the primary tumour is impossible to evaluate using ERUS techniques and will inevitably lead to understaging. According to the published meta-analysis, ERUS does not perform any differently to either CT or MRI. However, this analysis limited assessment to T and N staging only and did not take into account the important prognostic variables that should also be assessed by imaging, namely depth of extramural spread in millimetres, relationship of tumour to the mesorectal fascia and extramural vascular invasion [13]. It has been suggested that the published literature overestimates the performance of ERUS in staging rectal cancer with median T stage accuracy of 69 % and N stage of accuracy 65 % for studies of >300 subjects—again there is no published data on the ability of ultrasound to stratify prognostic groups [14]. Despite many expectations, ERUS has not proven to be effective in staging ERC, as preoperative staging by these methods does not influence local recurrence or tumour-specific survival [15]. The UK transanal excisional microsurgery (TEMS) registry group reported that ERUS inaccurately staged rectal cancer in 44.8 % out of 165 patients who underwent TEMS for ERC, and no significant difference was found in the depth of TEM excision or R1 rate between the patients who underwent ERUS before TEM and those who did not (*p* = 0.73) [16]. As an assessment tool for potentially significant polyps, ERUS with an elastography algorithm could potentially be useful for differentiating malignant transformation within rectal adenomas; a study by JER Waage et al. showed that strain elastography could assess tissue hardness which enabled distinction between benign adenomas vs invasive adenocarcinomas with 0.86 (0.66–0.96) specificity and 0.94 (0.88–0.97) accuracy when compared to final histopathology [17].

## CT

The overall multidetector CT accuracy of T staging is around 86 % [18]; however, its sensitivity to predict relationship of the tumour to CRM is less than 50 % [19], making the method unreliable for clinical practice. Invasion of the adjacent structures may be difficult to assess due to lack of fat plane between the tumour, mesorectal fascia and adjacent structures anteriorly or in the lower rectum.

For lymph node assessment, size is most commonly used CT criterion for differentiating benign from malignant lymph nodes; however, Bipat et al. meta-analysis showed that CT had 55 % sensitivity and 74 % specificity for predicting lymph node involvement in rectal cancer patients [13].

Nevertheless, published data suggests that such risk factors of tumour spread as mucinous content and presence of EMVI could be identified on CT scans. Heterogeneous enhancement and presence of areas of hypoattenuation are more frequently identified within the mucinous tumour vs non-mucinous [20]. It has been shown that CT allows visualisation of EMVI characterised by nodularity and expansion of perirectal vessels [21], and in colon cancers, it can be used to stratify good vs poor prognosis using depth of extramural spread of >5 mm as a definition for poor-risk tumours [22, 23].

## MRI

MRI has become the optimal modality for the local staging of primary and recurrent rectal cancer. There are several advantages over alternative techniques: it enables to stratify tumours depending on the presence of several high-risk features that are not limited to T and N stage (CRM and EMVI status, depth of extramural invasion, presence of discontinuous extramural vascular spread/deposits, presence of mucin and grade of tumour regression to preoperative treatment). These have all been proven to influence disease-free and overall survival rates in prospective multicentre studies. In recurrent rectal cancer, MRI allows the delineation of tumour extent within the pelvic compartments, can assess the pattern and mode of local recurrence and predict resectability of the disease.

Specific imaging parameters should be followed to achieve optimal results of the MRI technique. Good quality high-resolution scan can be achieved if the following technique criteria are satisfied (Table 2):

- Small FOV (not more than 160 mm) coronal, sagittal and axial slices
- Slice thickness (no more than 3 mm and voxel size less than 1.5)
- Correct scan plane alignment (perpendicular to the rectal wall at the level of tumour advancing edge)

**Table 2:** Optimal MRI protocol for achieving high-resolution scans.

Sequence	Sag TSE T2	Axial TSE T2	Axial TSE T2 high-resolution	Cor TSE T2
TR	3961	4018	5362	5362
TE	125	80	100	100
TSE factor	23	20	16	16
FOV/RFOV	250/100 %	300/100 %	160/90 %	160/90 %
Slice thickness/gap	3/0.4	5/1	3/0.3	3/0.3
NSA	4	2	6	6
Matrix	320/512	256/512	256/256	256/256
Sat bands	Ant/Sup	None	None	None
Acquisition time	6.00	3.28	7.35	7.35

*TR* repetition time, *TE* echo time, *TSE* turbo spin echo, *FOV* field of view, *RFOV* reduced field of view

The voxel and FOV size increase will cause loss of resolution, so that assessment of tumour morphological characteristics becomes challenging. Incorrect imaging plane incline usually leads to tumour overstaging.

Intravenous contrast to assess primary rectal cancer or recurrence is not recommended as there is no evidence that its use improves accuracy or gives additional prognostic staging information.

### Primary Tumour Staging

#### T Stage

On high-resolution MRI scans, rectal wall anatomy is clearly depicted so depth of tumour invasion can be correctly assessed. Not only is extension into the mesorectum can be evaluated but also tumour spread within the layers of the bowel wall. Therefore, MRI enables to identify patients with early rectal tumours eligible for organ-sparing surgical treatment (local excision) and those with high-risk tumours, when preoperative treatment is mandatory.

#### Early Rectal Cancer Staging

All rectal cancers apart from mucinous tumours demonstrate intermediate signal intensity (SI), whereas the submucosal layer shows hyperintensity and the muscularis propria (MP) low-intensity MR signal. The presence of a hyperintense stripe between the tumour and muscularis at the level of tumour advancing border (not the rolled edges) should be interpreted as full or partial preservation of submucosa and indicates a likely T1sm1/2 at most; on the contrary, absence of preserved submucosa but unaffected MP suggests T1sm3/T2 (with partial invasion of the muscularis)—from the clinical point of view, there is no need in differentiating these two stages (sm3/T2); if no macroscopic invasion of the mesorectal fat is present and no low signal intensity line identified between intermediate SI tumour and hyperintense mesorectal, tumour should be staged as T2 (with full thickness of MP invasion)/T3a (<1 mm tumour spread beyond the muscularis propria)—prognosis and outcomes of patients with these T stages are no different, so their distinction is unnecessary.

#### Advanced Rectal Cancer Staging

Macroscopic spread of tumour beyond the MP associated with worsening prognosis. Tumour spread <5 mm is associated with identical prognosis as T2 tumours provided that there is no CRM involvement by tumour. More advanced penetration into the mesorectum (T3c-d disease) is associated with increasing rates of disease recurrence [24, 25]. Subclassification of T3 stage based on depth of tumour spread into the mesorectum should be used, while MRI staging is performed as MRI has been proven to be accurate in substaging T3 tumours with measured equivalence to histopathology [26].

### Circumferential Resection Margin

The outermost boundary of the mesorectum is defined by the mesorectal fascia (MRF), which acts as a barrier to tumour spread and demarcates the CRM during surgical excision. MRI has been proven to accurately identify potential distance from tumour to CRM within 1 mm, which is recognised by pathologist as a clear margin [2, 27–29]. Results of the prospective MERCURY Study showed that specificity of MRI for predicting pCRM is 92 % [30].

According to the TNM classification system, tumours invading the mesorectal fascia are not T4—therefore, the predicted MRI CRM status must always be reported in addition to the TNM. A measured distance of 1 mm or less from the primary tumour, tumour/vascular deposit, or invaded extramural vessels to the MRF is one of the major prognostic factors for local recurrence, poor disease-free survival and poor overall survival and should always be stated in the MRI reports.

### N Stage

Nodal staging is of questionable importance as a predictor for local recurrence in patients receiving radical surgery for primary tumours; however, it still should be assessed if local excision is considered. Some authors continue to suggest using size criteria when determining the nature of mesorectal nodes; however, there is no rational basis for this and all published pathology data indicates that there is no relationship between the size of a lymph node and its likelihood of malignancy [31]. In a histological survey of over 12,000 lymph nodes from rectal cancer, specimens showed a considerable size overlap between normal, reactive and malignant nodes [31]. A prospective study where nodes from in vivo MRI, MRI specimens and pathology specimens were matched showed that there was no useful size cutoff for predicting nodal status [32]. Rather than size, it is the morphologic MR features such as presence of heterogeneous MR signal and irregular borders of lymph node capsular that are more reliable in differentiating nature of changes within nodes, and this has been prospectively validated in several studies [32, 33].

### EMVI

MRI EMVI defined as tumour signal within the vasculature outside the muscularis propria has been proven to correlate with histopathology findings [4] and survival outcomes [34]. On high-resolution MRI, EMVI is demonstrated as expansion of veins due to tumour signal present within them. It is an independent risk factor for metastatic disease, and its presence in low rectal cancers is also associated with a risk of CRM involvement and consequent local recurrence [35, 36••].

### Recurrent Rectal Cancer

Assessment of tumour resectability is one of the crucial things in recurrent rectal cancer patients. Offering the best soft tissue contrast compared to the rest of the available imaging modalities MRI enables evaluating relationship of recurrent tumour to important landmarks—pelvic viscera, peritoneum, bones, nerves, muscles, fasciae and ligaments. It has been highlighted that accurate delineation of disease extent in relation to pelvic compartments is essential to plan the most appropriate treatment for this group of patients [6•].

### Tumour Response Assessment

After preoperative CRT, up to 30 % of patients will develop complete pathological response defined as no tumour identified on the final histology examinations. Modified Mandard grading system of tumour response was tested in MERCURY study and has been proven to be a reliable tool for assessing tumour response in rectal cancer [37••]. This grading scale is based on qualitative assessment of intermediate signal vs low signal (the latter considered as fibrosis and the former as residual disease) within the treated tumour. MR TRG 1–2 can be described as low signal intensity scar or low density fibrosis with no evident macroscopic intermediate signal intensity within it, mrTRG3 dominant fibrosis outgrowing the tumour mass and mrTRG 4–5 predominantly intermediate signal intensity with minimal or no signs of fibrosis. MERCURY experience showed that patients with complete and near complete response (mrTRG 1–3) have better prognosis, disease-free and overall survival compared to poor responders [38].

Some studies suggest using DW-MRI in order to increase accuracy of tumour response assessment and specificity for selecting patients eligible for deferral of surgery trial, proposing that patients showing residual diffusion restriction on high *b* value DW images should not be offered watch and wait policy [39, 40]. However, there is no published data proving the efficacy of applying DWI in response assessment. Thus far, no publication has proven any added value of DWI compared against mrTRG, ypT, ymrT, in assessing response, overall and disease-free survival rates. A recent analysis has shown that only 32 % of patients with imaging complete response (mrTRG1-3) would have been included in the protocol if diffusion-weighted criteria had been applied, yet no difference in the rate of tumour regrowth was identified amongst the 18 patients from DWI group (33.3 %) compared to 56 patients in the mrTRG 1–3 group (37 %) (*p* = 0.82) [41]. The data indicates that DW-MRI not only underestimates patients with sustained complete response and does not improve specificity compared with mrTRG.

## PET/CT

The role of an imaging modality that provides a combination of functional information (18F-FDG-PET) and morphologic information (CT) has not yet been defined for rectal cancer staging. This method is not recommended in the international standards of rectal cancer patients’ management or guidelines for local spread assessment [7]. It has been shown that PET/CT detects colonic abnormalities larger than 13 mm in diameter with 90 % accuracy [42]; however, the specificity for differentiating hyperplastic benign polyps from primary colorectal cancers is reported to be 43 % and thus the application of this technique is uncertain [43].

The high cost and lower availability of PET are not the only disadvantages of the method. It has been shown to be inaccurate in differentiating changes within the mesorectal and pelvic lymph nodes [43, 44], it is also known to be insensitive for assessing mucinous tumours and there is no data suggesting that it could improve patient selection with complete response [45–47]. It is known that up to 25 % of 18F-FDG-uptake occurs in metabolically active but non-malignant tissues, such as granulation or inflammation [48, 49]. There are several studies where response monitoring was performed and compared with morphological response, and it has been demonstrated that the reduction in SUVs was significantly greater in (histopathologically confirmed) responders than in nonresponders [50, 51]; however, there is no consensus on the SUV reduction rates needed to predict complete response.

A meta-analysis published by Huebner et al. included 11 studies and 577 patients showed that FDG-PET had 97 % sensitivity and 76 % for detecting recurrent colorectal cancer [52]. However, Suga and colleagues reported that prevalence of PET-positive cases in rectal cancer patients was higher with an increase in carcinoembryonic antigen (CEA) levels (41 % PET positivity for CEA level of 5–10 vs 83 % with CEA level >50) [53]. Therefore, a rising CEA level, equivocal findings on other imaging, should be considered an indication for PET/CT in patients with known or suspected recurrent colorectal cancer[54••].
